# Draft Genome Sequences of Two Isolates of the Plant-Pathogenic Fungus *Neonectria ditissima* That Differ in Virulence

**DOI:** 10.1128/genomeA.01348-15

**Published:** 2015-11-19

**Authors:** Cecilia H. Deng, Reiny W. A. Scheper, Amali H. Thrimawithana, Joanna K. Bowen

**Affiliations:** a The New Zealand Institute for Plant & Food Research Limited (PFR), Auckland, New Zealand; b The New Zealand Institute for Plant & Food Research Limited (PFR), Havelock North, New Zealand

## Abstract

*Neonectria ditissima* is the causal agent of apple canker. Here, we present the draft genome sequences of two isolates of *N. ditissima* that differ in virulence. Comparative genomics will enable pathogenicity determinants to be identified in this plant-pathogenic fungus.

## GENOME ANNOUNCEMENT

*Neonectria ditissima* (Tul. & C. Tul.) Samuels & Rossman (synonym *Neonectria galligena*) is a fungal pathogen causing canker disease in many broad-leaved trees, including iconic Northern Hemisphere forest and specimen trees and the horticultural genera *Malus* (apple) and *Pyrus* (European pear [1, 2]). In these fruit trees, the disease in apple (European canker) is especially severe and is of importance to growers across both the Northern and Southern Hemispheres, although it is particularly damaging in mild, wet climates (3, 4). Apple canker is currently controlled by pruning and fungicides. Different apple varieties show different susceptibilities to canker, hinting at the possibility of breeding a highly tolerant or resistant cultivar (5). Variation is also seen in the pathogen population. At the level of host range determination, *formae speciales* probably exist within *N. ditissima sensu lato* (1), and variations in virulence (6) and conidial morphology (7) are seen within the populations of *N. ditissima* able to infect apple. Comparative genomics of isolates varying in virulence against apple may provide details on which pathogenicity determinants are essential for virulence and pathogen fitness.

Thus, two single-ascospore isolates of *N. ditissima* from New Zealand were selected for whole-genome sequencing: RS305p, isolated from *Malus x domestica* cv. ‘Brookfield,’ a naturally occurring isolate with very low virulence; and RS324p, isolated from *M. x domestica* cv. ‘Golden Delicious,’ a virulent isolate (6). For each of the isolates, genomic DNA was extracted from axenic *in vitro*-grown fungal material. Two paired-end libraries (insert sizes, 180 bp and 400 bp) and two mate-pair libraries (5 kb and 10 kb) were constructed for each isolate and sequenced on the Illumina HiSeq 2000 platform. The genomes were estimated to be 45 Mb in size, based on the average size of related *Fusarium* genomes (8), giving a sequence depth >200× for both isolates. The *de novo* assembly was carried out using Platanus-1.2.1 (9) and scaffolded with SSPACE version 2.0 (10). Gap filling was performed with GapCloser version 1.12 in the SOAP Tools package (http://soap.genomics.org.cn/soapdenovo.html). For isolate RS305p, the assembled draft genome was 43.56 Mb in size, consisting of 189 scaffolds, with an *N*_50_ metric of 1.17 Mb and a longest scaffold of 5.5 Mb, while the genome of isolate RS324p was 44.95 Mb in size, consisting of 172 scaffolds, with an *N*_50_ metric of 1.81 Mb and a longest scaffold of 4.05 Mb. To check the completeness of the genome assemblies, CEGMA version 2.4.010312 (11) was run, and both assemblies were >95.5 percent complete. All of the *N. ditissima* (*n* = 107) and *N. galligena* (*n* = 83) sequences available from NCBI were correctly assembled in both genomes. Using AUGUSTUS version 3.0.3 (12) with the *Fusarium* model, 12,561 protein-coding genes were predicted for both isolates, and 471 and 461 tRNAs were detected using tRNAscan-SE in the RS305p and RS324p genomes, respectively (11). Among the predicted proteins, a search of the Pathogen-Host Interactions database (PHI-base [13]) identified those with similarity to two effectors (avirulence determinants) and six proteins with a mutant phenotype of hypervirulence in both isolates.

### Nucleotide sequence accession numbers.

This whole-genome shotgun project of *N. ditissima* has been deposited at DDBJ/EMBL/GenBank under BioProject PRJNA285413 with the accession no. LDPK00000000 for isolate RS305p and LDPL00000000 for RS324p. The version described in this paper is version 1.0.
